# Development of a control task for clarifying the neural mechanisms underlying tool-use behavior in rats (*Rattus norvegicus*)

**DOI:** 10.1016/j.mex.2019.11.022

**Published:** 2019-11-27

**Authors:** Akane Nagano

**Affiliations:** Organization for Research Initiatives and Development, Faculty of Psychology, Doshisha University, Japan

**Keywords:** Control task of tool-use task for clarifying the neural mechanisms underlying tool-use behavior, Rats, Rodents, Tool-use behavior

## Abstract

Many studies on non-human animals have attempted to investigate the neural mechanisms underlying tool-use behavior. However, previous studies showed considerable non-tool-use-specific differences between tool-use and control tasks. The purpose of the present study was to develop a control training task for studies that investigate the neural mechanisms behind tool-use in rodents. Eight rats were subjected to control tasks which excluded tool-use-specific factors and consisted of training for hook-pulling and hook-choice tasks, as well as tool-choice tests which included tool-use specific factors and were similar to those in a previous study on rats. With the exception of one rat, the results of the hook-choice training showed that the previous study and the present study had similar difficulty levels. In the tool-choice tests of the present study, rats did not choose the functional tools over the non-functional tools when there was no contradiction between their appearance and functionality, which contrasted with the previous study on which this study was based on. These results suggest that the training task that excludes tool-use-specific factors can be appropriately utilized as a control task for studies investigating the neural mechanisms behind tool-use in animals and, potentially, in humans.

• Hook-choice training without tool-use-specific factors can be performed as a control task.
• Prior tool-use training improved rats’ performance in experimental tests.
• Control task for rodents allows investigation of the neural mechanisms of tool-use.

## Highlights

• Hook-choice training without tool-use-specific factors can be performed as a control task.
• Prior tool-use training improved rats’ performance in experimental tests.
• Control task for rodents allows investigation of the neural mechanisms of tool-use.

## Graphical abstract

**Figure:**
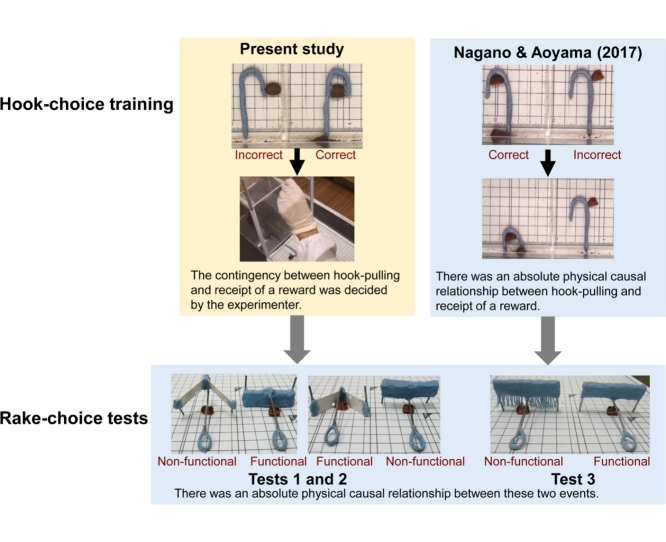

**Specification table**

**Table:** 

Subject Area:	Psychology
More specific subject area:	Physiological Psychology
Method name:	Control task of tool-use task for clarifying the neural mechanisms underlying tool-use behavior
Name and reference of original method:	Tool-use task A. Nagano, K. Aoyama, Tool-use by rats (Rattus norvegicus): tool-choice based on tool features, Anim. Cogn. 20 (2) (2017) 199–213.
Resource availability:	*N/A*

## Method details

### Background

Previous studies on humans [1] and non-human animals [2] have attempted to examine the neural mechanisms underlying tool-use behavior. One study on rodents reported that tool-use behavior induced adult neurogenesis in the dental gyrus of the hippocampus [3]. Another study on monkeys reported a significant increase in cerebral blood flow in specific areas when performing tool-use behavior [4]. Both of these studies reported that cerebral changes were not induced by control tasks, but were specific to tool-use tasks. In these studies, the subjects were required to manipulate a rake-shaped tool in order to obtain a food reward which was placed beyond their reach [3,4]. In the control condition of the rodent study, degus (*Octodon degus*) were tasked with simple spatial learning in a radial arm maze [3]. The control condition of a primate study was a simple-stick manipulating task whereby Japanese macaques (*Macaca fuscata*) were rewarded with food if they swung a simple-stick fixed to a plate with a universal joint [4].

However, one cannot conclude from these studies that the cerebral changes were tool-use-specific, since there were differences between the tool-use tasks and the control tasks in both studies [3,4]. In other words, non-tool-use-specific differences (e.g., the differences related to the simple movement required to obtain rewards) between the tool-use and control tasks may have induced the cerebral changes, rather than the tool-use behavior itself. Therefore, control tasks which are as similar to tool-use tasks as possible, but exclude tool-use-specific factors are required to reveal whether the cerebral changes were indeed induced by tool-use behavior or specific brain regions that contribute to the behavior.

This study aimed to develop a control task for studies investigating the neural mechanisms behind tool-use tasks in rodents. Visalberghi and Tomasello [5] implemented tool-use tasks to investigate the physical causal understanding in non-human animals. They suggested that animals can comprehend how the antecedent event “A” (the cause) produces the consequent event “B” (the effect), and not just that event B always occurs after event A. In the present study, rats were subjected to a control training task which excluded tool-use specific factors but was similar to tool-use tasks used in previous studies on rats (*Rattus norvegicus*) [6] and primates [7].

This was followed by tool-use training, where tool-use-specific factors were included. There was an absolute physical causal relationship between event A, whereby the subject placed a tool behind some food and pulled it towards them, and event B, where the food approached the subject. Subjects had opportunities to learn the relationship between these two events [6].

In the control training, tool-use-specific factors were excluded. There was no absolute physical causal relationship between event A, manipulation of an object with a similar appearance and placement to the tool used in the tool-use training [6], and event B, where the food approached the subject. The contingency between these two events was manipulated by the experimenter.

Procedures in this study were kept as similar as possible to those in a previous study [6]: the experimental box was identical, subjects performed the same number of trials in each training session, and similar tool and food movements occurred after subjects pulled the tool. My hypothesis was that performance in the tool-choice tests that included tool-use-specific factors would be lower than those in the study by Nagano and Aoyama [6].

### Subjects

Eight experimentally naïve three-month-old male Brown-Norway rats (subject numbers: BN21–BN28; Shimizu, Kyoto, Japan) were individually housed in wire cages. On the last day of free-feeding, the rats weighed an average of 253.38 g (*SD* = 9.28). During training and testing, rats were maintained at around 85 % of their free-feeding weight. However, all rats could gain approximately 10 g/month. The animal room was maintained under a 12 -h light/dark cycle (light phase 8:00–20:00). All training and testing sessions were conducted during the light phase. All procedures and treatments were approved by the Doshisha University Animal Experiment Committee, and were conducted in accordance with guidelines established by the Doshisha University Ethics Review Committee.

### Apparatus

Experiments were performed in an experimental box that was almost identical to one used in the previous study on rats [6], excluding the addition of a sliding door. The experimental box (outer dimensions: 21.0 cm wide ×21.0 cm long ×25.6 cm high) was made from transparent acrylic boards. The box was placed on a desk in the experimental room.

The transparent sliding doors (21.0 cm wide ×32.0 cm high ×0.3 cm thick), which the experimenter could open/close by hand, were mounted on the front of the box. One of two kinds of sliding doors (one without holes and one with a square hole) was always placed in front of the experimental box. The door with a square hole in its upper portion was used to offer food rewards to the rats by hand (Supplementary Video 1). The square hole of the door (1.5 cm wide ×1.5 cm high) was centered horizontally and located at a height of 21.0 cm.

An experimental board, on which the tools, other objects, and food were presented, was positioned in front of the sliding door. The board consisted of a white cutting mat (23.0 cm wide ×32.0 cm long ×0.3 cm thick, Sekisei Co., Ltd., Osaka, Japan) and a transparent acrylic board (23.9 cm wide ×33.5 cm high ×0.5 cm thick), with the cutting mat placed on the acrylic board. A transparent acrylic board (0.5 cm wide ×29.0 cm long ×1.0 cm high) was placed as a partition in the center of the experimental board while conducting all training and tests, except during the shaping phase (hook-pulling training). This partition prevented the two hooks positioned for the experiment from coming into contact with each other. Black drawing paper was laid underneath the experimental box.

**Fig. 1:**
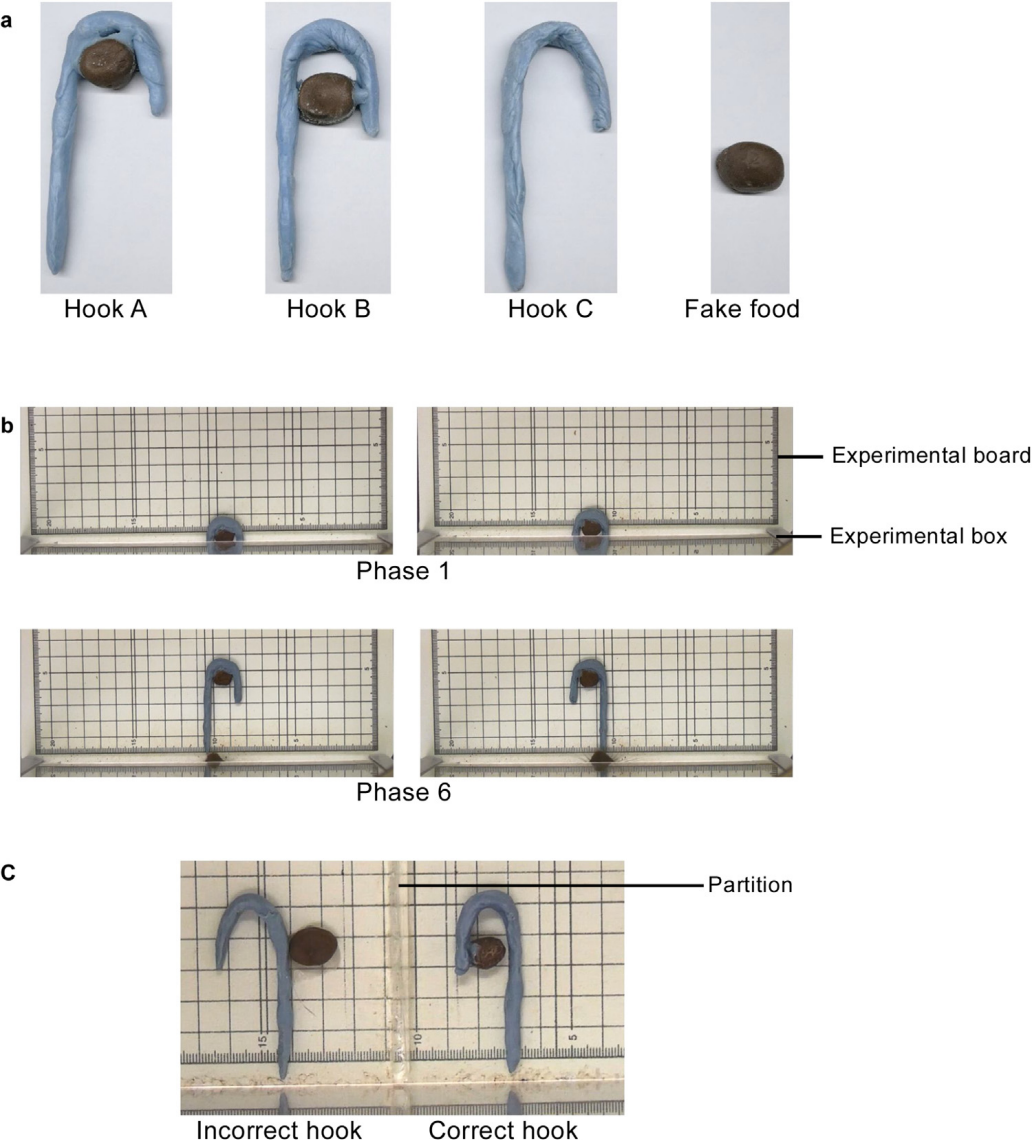
The hooks, fake foods used, as well as their arrangements in the hook-pulling and the hook-choice training. (a) Hooks A and B (correct hooks), Hook C (incorrect hook) and fake food. (b) Arrangements of Hook A and the fake food in Phases 1 and 6 of the hook-pulling training. (c) Example of the hook arrangement with their fake foods in the hook-choice training. The same 12 arrangements of hook and fake food as conducted by Nagano and Aoyama [6]. A partition at the center of the experimental board which prevented the hook that the rat pulled from contacting another hook.

A total of 73 hook-shaped objects (19 Hooks A, 18 Hooks B, and 36 Hooks C) and 73 fake foods were used for training (Fig. 1a). The shapes and colors of the objects and fake foods were very similar to those used by Nagano and Aoyama [6]. Hooks were made of aluminum (Hooks A and B, maximum 2.3 cm wide ×6.0 cm long × maximum 1.0 cm high) or iron wire (Hook C, maximum 2.3 cm wide ×6.0 cm long × maximum 1.0 cm high) and covered with resin for dental use (Ostron II Blue, GC Corporation, Tokyo, Japan). All hooks were light blue. Hook A and B together with its corresponding fake food weighed as much as each Hook C without the fake food, with a weight range of 2.90–3.10 g. The fake food (maximum 0.8–1.5 cm wide × maximum 0.7–1.0 cm high) was made from a small rounded aluminum foil which was covered with a mixture of powder (31.09 g) and liquid resin, and 5.52 g of cocoa powder (Kyoritsu-foods Co., Ltd., Osaka, Japan). The fake food used in all instances were designed to imitate to a piece of a chocolate-flavored loop cereal (Ciscorn Sakusaku Ring, Nissin Cisco Co., Ltd., Osaka, Japan) used as the food reward in the study by Nagano and Aoyama [6]. In the training, three kinds of hook-shaped objects were used (Hooks A, B, and C) and as well as 36 fake foods that were not attached to the hooks. The 73 hooks consisted of 37 hooks (Hooks A and B) for the correct options and 36 hooks (Hooks C) for the incorrect options (Fig. 1a). One of 37 hooks (one Hook A) for the correct options was used only in the first training session. Hooks A and B had fake food glued to their interior. In contrast, Hook C did not have fake food attached to it. Hooks A, B, and C and the fake food, independent of the hooks, were designed to imitate those of the hook-choice training in the study by Nagano and Aoyama [6]. The fake food was glued to Hook A so that it stayed in contact with the hook bend, while a 1.0 cm space was maintained for Hook B, between the fake food and the hook bend. In contrast, no object was glued to the interior of Hook C, as the food and hook were presented separately.

In the rake-choice tests, four different rake-shaped tools (Rakes A, B, C and D) were used. These four rakes were identical to those in the tests of Nagano and Aoyama [6]. Rake A had a blade made of acrylic board covered with resin for dental use, and Rake B had a blade made of a white l-shaped metal fitting with the same resin. Rakes A and B could be used as either functional or non-functional rakes for each test because they had a vertical wire glued onto the end of each blade which pointed upwards. This rendered the rakes non-functional when placed upside down due to the empty space beneath the blade. While Rake C initially appeared to be non-functional, this was, in fact, functional for obtaining food since there was a transparent acrylic plate glued between the two wires under the blade. In contrast, while Rake D initially appeared to be functional for obtaining the reward, in reality this was non-functional since 39 light blue embroidery threads were glued to the bottom of the blade at equidistant intervals. A vertical wire pointing upward was glued to each blade end of Rakes C and D, and a handle made of wire and resin was glued to the center of blade of each rake. Additional details are described in a previous study [6].

During the training and testing sessions, the subject’s behavior was recorded by a video camera (Panasonic, Japan, HDC-TM30) mounted above the experimental box. The experimenter sat in front of the box, observed the subject’s behavior, and performed the following behavioral procedures.

### Procedure

#### Habituation

Before the training phase, rats were handled for 5 min per day for 5 days. The feeding restriction was introduced to control the subject’s weight on the third day of handling. From the third day on, each rat was habituated to the food reward by receiving chocolate-flavored loop cereal in its cage for 3 days.

#### Trainings

The training consisted of hook-pulling training (40 trials per session) and hook-choice training (36 trials per session). During these training sessions, the previously discussed tool-use-specific factors were excluded; i.e., the contingency between hook-pulling behavior and receipt of a reward was decided by the experimenter.

With the aim to set the experimental conditions such that the contingency was decided by the experimenter and the appearances of the hooks and the rewards were similar between the tool-use [6] and control trainings, fake foods were used in the control training to exclude absolute physical causal relationships. In contrast, if real foods had been used for the options, the rats always could have obtained the rewards by themselves by pulling the correct hooks, thereby establishing absolute physical causal relationships between the events. The experimenter offered a real food to the rats as a reward when they chose the correct hooks to provide the rats feedback as to which option was correct.

#### Hook-pulling training

For the hook-pulling training, the rats learned to pull Hook A to a position in which the fake food, which was glued to the hook, entered the experimental box. The trial ended either when the rat pulled the hook to the position in which the fake food entered the box (a successful trial) or when 60 s had passed without success (a failed trial). For successful trials, the experimenter offered the food reward by hand through a small hole in the door of the box immediately after the glued fake food entered the box, and then retrieved the hook. For failed trials, the experimenter retrieved the hook when 60 s had passed.

The sliding door of the box with a square hole was kept open throughout the task so that the space between the lower part of the door and the surface of the experimental board was 1.7 cm. At the beginning of the session, the rat was placed in the box. The trials started when the experimenter placed Hook A at the center, in a defined position on the board, depending on the phase (Fig. 1b).

The distance between the hook and the rat was progressively increased, and these distances were divided into six phases (Fig. 1b; Supplementary Video 2). The distance was increased by 1.0 cm every time the rat fulfilled the criterion of the previous phase. In Phase 1, Hook A was placed such that the distance between the fake food and the sliding door was 0 cm. At the beginning of Phase 1, their hook-pulling behavior was shaped by the method of successive approximations. Specifically, if the rats touched the hook with its left paw, right paw, nose, or mouth, the experimenter offered a food reward at the beginning of this phase. In the next step, if the rats pulled the hook with their paw or mouth, the experimenter offered a reward. After the rats began to consistently pull the hook in this phase, the experimenter offered a reward only when they pulled the hook to the position in which the fake food entered the box (a successful trial). From Phase 1 to Phase 5, five successful trials resulted in advancement to the next phase, and the same criterion was utilized for the last phase (Phase 6). This hook pulling training continued for every rat until it attained the criterion of Phase 6. The training for rats to pull the hook to the defined position took nine days.

Two hook arrangements were utilized during all phases (Fig. 1b). Each arrangement was used in half of the trials each day in pseudo-randomized order.

#### Hook-choice training

For the hook-choice training, rats had to choose between a correct hook (either Hook A or B) and an incorrect hook (Hook C; Fig. 1a). They were trained to choose the correct hooks based on the spatial arrangements of the hook and the fake food. The movements of the food rewards in the corresponding training in Nagano and Aoyama [6] were reproduced using fake food in this training. The procedures were the same as those for the hook-choice training in the study by Nagano and Aoyama [6], except for the use of fake food, the number of hooks for the correct and incorrect options, and the method of presenting rewards to the rats after successful trials. If the rat pulled the correct hook, the reward was offered exactly like in the hook-pulling training (Supplementary Video 3). In contrast, if the rat pulled the incorrect hook, the experimenter retrieved the hooks and did not offer the reward.

In this training, the same 12 arrangements of hook and fake food by Nagano and Aoyama [6] was adopted. Each of the 12 arrangements was presented three times in a pseudorandom order in each session, and each daily experimental session consisted of 36 trials. For this training, 18 Hooks A and 18 Hooks B were used as the correct options, and 36 Hooks C and 36 fake foods independent of each hook, was used as the incorrect options. Each hook and the fake food was presented once in each session.

At the beginning of the session, the rat was placed in the box with the sliding door that had the square hole closed. The space between bottom of the door and the surface of the board was 1.7 cm. The sliding door was opened 3 s after the experimenter placed hooks and fake foods at the defined positions on the experimental board (trial start, Fig. 1c). For the correct options, Hooks A or B were simply placed on the board. For the incorrect options, Hook C and the fake food - which was adhered to the surface of the board with double-sided tape - was placed on the board simultaneously, with the fake food on the other side of the hook. Thus, the rats could not obtain the fake food even if they pulled Hook C, and the fake food did not move even upon contact with Hook C.

When the rat pulled either the correct or the incorrect hook by 2.0 cm, it was regarded as selecting the hook. If the rat selected the correct hook, the experimenter retrieved the incorrect hook and fake food immediately, offered the reward by hand through the square hole in the door, and then retrieved the correct hook before closing the door. If the rat selected the incorrect hook, the experimenter retrieved the correct hook immediately after, closed the door after 30 s, and did not offer any food rewards. Immediately before the door was closed, the experimenter also retrieved the incorrect hook and the fake food. If the rat made no selection after 60 s had passed, the experimenter retrieved both the correct and incorrect options and closed the door. When the rat selected the correct hook and pulled it so that its fake food entered the box within 60 s, it was recorded as a successful trial. When the rat chose either the incorrect hook, or the correct hook without pulling it to a position whereby the fake food entered the box, it was recorded as a failed trial. Moreover, when the rat chose neither the correct nor the incorrect option within 60 s, it was recorded as a no-choice trial. This hook-choice training continued until each rat had attained the criterion of 30 or more successful trials for two consecutive sessions. One rat (BN22) did not attain the criterion within 83 sessions, and the rake-choice tests for this rat was conducted one day after session 83 of the hook-choice training.

#### Rake-choice tests

The rake-shaped tools and procedures for the rake-choice tests were identical to those in the study by Nagano and Aoyama [6]. The rake choice tests included tool-use specific factors; i.e. the rats could directly obtain the food reward by pulling the correct one of two different rakes. The tests consisted of Tests 1, 2 and 3, each of which was conducted over one session. In Tests 1 and 2, there was no contradiction between the appearance and the functionality of the tools, (Supplementary Video 5) while there was a contradiction in Test 3 (Supplementary Video 6). Between each test, the rats were retrained with the hook-choice training for two sessions.

### Data analyses

During training, the daily success rate was calculated by dividing the number of successful trials by the total number of trials (36 trials/day). Data were analyzed using paired *t*-tests with a between-subject factor.

In rake-choice Tests 1, 2, and 3, the choice rate of the functional rake was calculated for each rat by dividing the number of trials in which each rat chose the functional rake by the number of trials in which they chose either the functional or the non-functional rake. Data were analyzed using two-tailed binomial tests for each rat and for all tests.

### Method validation

#### Hook-pulling training

All rats began pulling the hook consistently between 2 (BN25, BN28) to 8 sessions (BN27), and they attained the criterion in the hook-pulling training between 3 (BN28) and 9 (BN27) sessions. All rats sought the reward fallen on the floor of the experimental box, and ate this at the beginning of the training. They became to start eating the reward immediately after the reward fell on the floor of the box, or catch the reward directly with their mouth or paws.

#### Hook-choice training

**Fig. 2:**
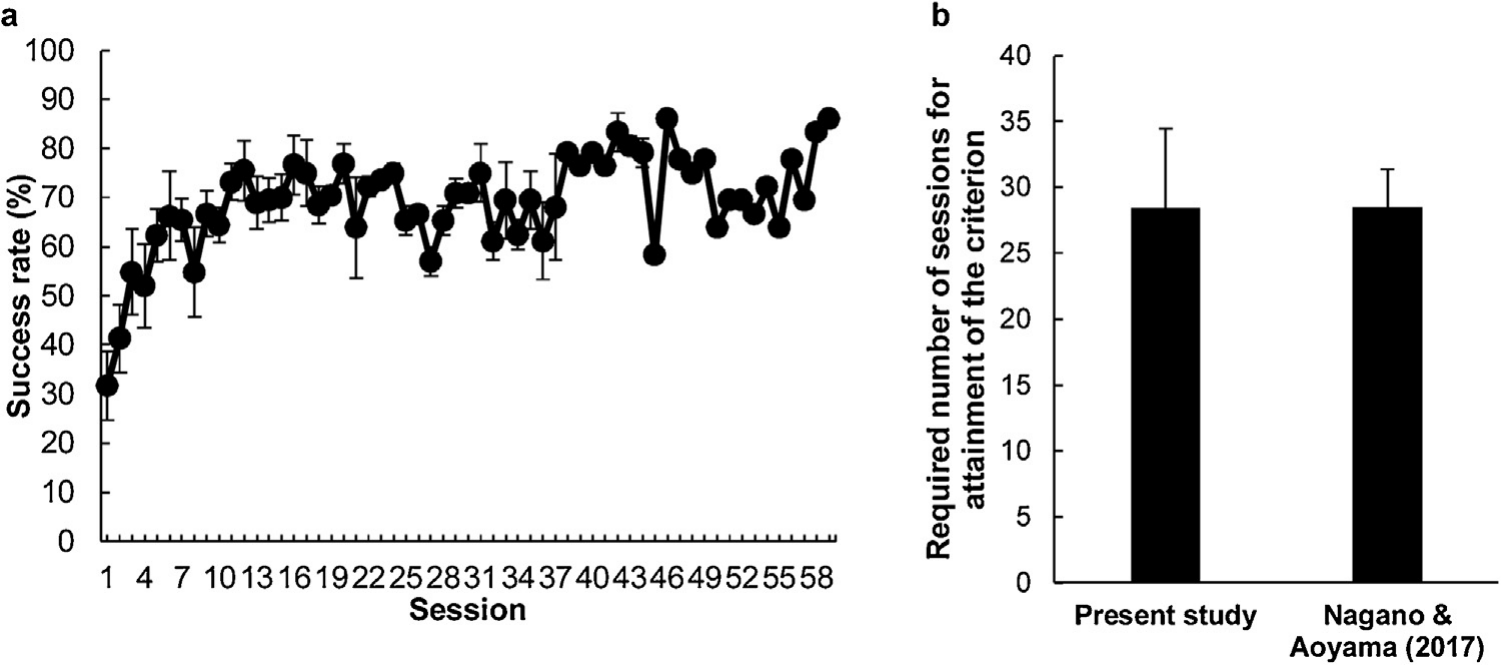
Performance during hook-choice training. (a) Average success rate in the hook-choice training. One rat (BN22) was excluded from this analysis. Error bars indicate standard error. The number of rats decreased as the session progressed. (b) The required number of sessions until each rat attained the hook-choice training criterion in the present study and a previous study [6]. One rat (BN22) was excluded from this analysis. Error bars indicate standard error.

**Fig. 3:**
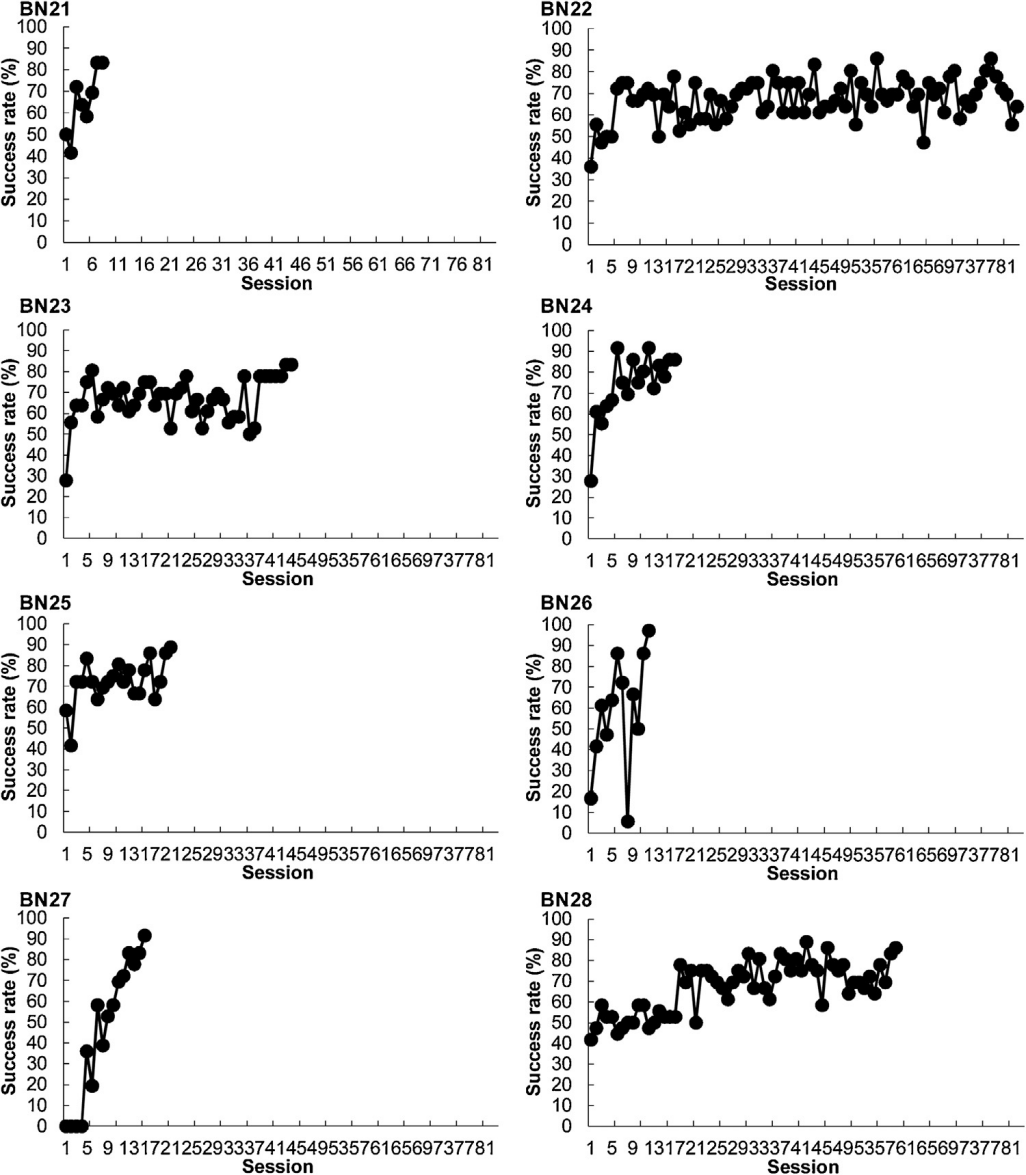
Success rates of individual rats (BN21–BN28) for the hook-choice training.

During hook-choice training, all rats except for one (BN22) attained the criterion between sessions 12 (BN26) and 59 (BN28) (Figs. 2a and 3 ). One rat (BN22) was therefore excluded from data analysis. A paired *t*-test between the rats in Nagano and Aoyama [6] and the present study with a between-subject factor revealed that there was no significant difference in the number of sessions until each rat attained the criterion between the two studies (*t* (13) = 3.43, *n.s.*, Fig. 2b), with the exception of one rat (BN22).

#### Rake-choice tests

**Fig. 4:**
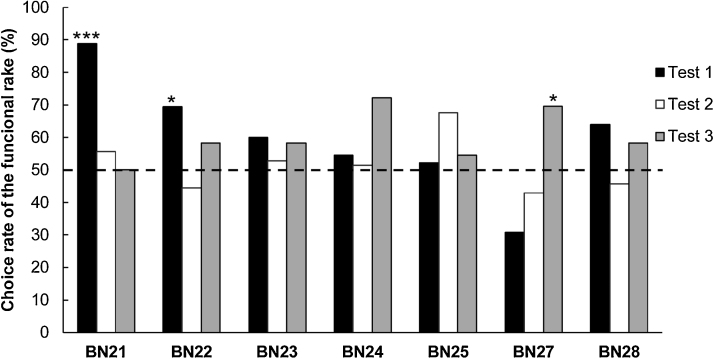
Individual choice rates of the functional rake in each rake-choice test. One rat (BN26) was excluded from this analysis. The broken line indicates chance level (* *p* <  0.05, *** *p* <  0.001).

In the rake-choice tests, one rat (BN26) did not choose any rakes except during a trial in Test 1, and was therefore excluded from data analysis. Results of two-tailed binomial tests for each rat across the three tests revealed that the rats were not able to choose the functional rakes in Tests 1 and 2 (Test 1: BN21: *p* <  0.001; BN22: *p* <  0.05; BN23–BN25, BN27, BN28: *n. s.*; Test 2: BN21–BN25, BN27, BN28: *n. s.*; Fig. 4), and only one rat (BN27) was able to choose the functional rake in Test 3 (BN21–BN25: *n. s.*; BN27: *p* <  0.05; BN28: *n. s.*; Fig. 4). In the identical tests performed by Nagano and Aoyama [6], all rats chose the functional over the non-functional rakes in Tests 1 and 2, but none chose the functional rake in Test 3.

## Conclusion

For the hook-choice training, non-significant difference in the number of sessions until each rat attained the criterion between the present study and the previous study [6] suggests that the training in these two studies had similar difficulty levels, and that the newly developed hook-choice training used in the present study, which excluded tool-use-specific factors, would be appropriate as a control for a tool-use task.

The results of the rake-choice tests in the present study and that by Nagano and Aoyama [6] indicate that the experience of tool-use training improved their performance in the tests. Some rats chose the functional over the non-functional rakes (Test 1: BN21, BN22; Test 3: BN27). However, no rats chose the functional rakes both in Tests 1 and 2, and thus it could be considered that these three rats did not choose the rake based on the functionality, but instead based it on preference.

Recently, many studies have attempted to reveal the neural mechanisms underlying tool-use behavior in humans by examining patients with brain damage as they undertake tool-use tasks [8]. Studies using rats would be useful to reveal these mechanisms since various experimental manipulations can be applied to rats, including microinjection of drugs and electrocautery lesions to specific areas of the brain [9]. For example, when a rat with a lesion in a specific brain area performs the same as a non-lesioned rat in the hook-choice training which excludes tool-use-specific factors (the present study), and when lesioned rats show worse performance than non-lesioned rats in the hook-choice training which includes tool-use-specific factors [6], it could be concluded that the lesioned area contributes to tool-use behavior. Thus, in this study, I propose a control task in rodents for the investigation of neural mechanisms of tool-use in animals and potentially humans.

## Declaration of Competing Interest

The author declare that there is no conflict of interest.
