# Supplementary material for: Development of a control task for clarifying the neural mechanisms underlying tool-use behavior in rats (Rattus norvegicus)
Source: MethodsX. 2019 Nov 27;6:2845–54. doi: 10.1016/j.mex.2019.11.022 (PMC6911953; doi:10.1016/j.mex.2019.11.022)
Supplement: Supplementary file 1 [file mmc1.docx]

**Supplementary materials**

**Supplementary Videos**

**Supplementary Video 1.** Demonstration of how a food reward was presented after the correct choice was made during hook-choice training. This behavior was not included in the data.

**Supplementary Video 2.** Examples of successful trials in Phases 1, 2, 5, and 6 of the hook-pulling training.

**Supplementary Video 3.** An example trial whereby a rat selected the correct hook during hook-choice training.

**Supplementary Video 4.** An example trial whereby the rat selected the incorrect hook during hook-choice training.

**Supplementary Video 5.** Examples in which the rat selected the functional and the non-functional rakes in the rake-choice Test 2.

**Supplementary Video 6.** Examples in which the rat selected the functional and the non-functional rakes in rake-choice Test 3.
